# Effect of Microstructure Refinement on the Corrosion Behavior of the Bioresorbable Mg-1Zn-0.2Ca and Mg-1Ca Alloys

**DOI:** 10.3390/ma15196749

**Published:** 2022-09-29

**Authors:** Olga B. Kulyasova, Ganjina D. Khudododova, Grigory S. Dyakonov, Yufeng Zheng, Ruslan Z. Valiev

**Affiliations:** 1 Institute of Physics of Advanced Materials, Ufa State Aviation Technical University, 12 K. Marx Str., 450008 Ufa, Russia; 2 Laboratory of Multifunctional Materials, Bashkir State University, 32 Zaki Validi Str., 450076 Ufa, Russia; 3 School of Materials Science and Engineering, Peking University, 5 Yi-He-Yuan Road, Hai-Dian District, Beijing 100871, China

**Keywords:** biodegradable Mg alloys, microstructure analysis, UFG structure, corrosion behavior, surface analysis, fine-dispersed particles

## Abstract

This paper presents a comprehensive study of the effect of the processing by high-pressure torsion (HPT) on the corrosion behavior in Ringer’s solution for two popular bioresorbable magnesium alloys—Mg-1Ca and Mg-1Zn-0.2Ca. Three states were studied for each alloy—the initial homogenized state, the as-HPT-processed state and the state after subsequent annealing at 250 and 300 °C. It is shown that HPT processing results in a very strong grain refinement in both alloys down to a mean grain size of about 210 nm for the Mg-1Ca alloy and 90 nm for the Mg-1Zn-0.2Ca alloy, but their corrosion resistance values differ significantly (by an order of magnitude). The conducted precision scanning electron microscopy (SEM), transmission electron microscopy (TEM) and X-ray diffraction studies demonstrate that such a difference in the corrosion behavior is conditioned by a difference in the morphology and origin of the nano-sized particles of second phases, as well as by a change in the electrochemical properties of the “particle–α-Mg” pair. The obtained results are discussed from the perspective of the innovative applications of biodegradable Mg alloys for the manufacture of advanced medical implants and products.

## 1. Introduction

Among current biodegradable metals, magnesium (Mg) and its alloys are the most promising for the use in various types of devices for the internal fixation of bone fragments in orthopedics [1,2,3]. Mg is an essential element in the human body: an adult person consumes about 300–400 mg per day. In addition, Mg has an elastic modulus and specific density close to those of human bones [3,4]. However, a too-fast dissolution of Mg in a physiological environment leads to a loss of mechanical integrity, which hinders its clinical application [5]. Therefore, it is very important to control the dissolution rate of Mg. As it is known, the corrosion of Mg can be described as the electrochemical reaction Mg + 2 H_2_O = Mg(OH)_2_ + H_2_ in an aqueous environment with the formation of magnesium hydroxide and hydrogen gas [6]. Alongside with biochemical reactions, the presence of cyclic loads may accelerate the corrosion of Mg even further. For example, a cardiovascular stent experiences a cyclic load due to the heartbeat, while an orthopedic implant experiences a load due to walking, running and regular body movements [7,8]. To increase the mechanical properties of Mg, Mg-Ca alloys were developed [9]. Calcium is well compatible with the human body and promotes bone tissue regeneration [3,9,10]. However, the fact that the corrosion properties of Mg-Ca alloys are not high, as well as the presence of the eutectic phase that causes alloy embrittlement, stimulated the design of Mg-Zn-Ca three-component alloys.

Zinc does not harm the human body and is considered an essential element for human health. Mg-Zn-Ca alloys exhibit an enhanced corrosion resistance and good strength properties [3,11]. For instance, a number of studies have shown that the addition of Zn in Mg-Ca alloys improves the corrosion resistance and increases hardness due to the formation of the Ca_2_Mg_6_Zn_3_ ternary phase [12,13,14,15,16].

Especially interesting are the studies of the properties of these Mg alloys with an ultrafine-grained (UFG) structure, where the grain size in the nanometric range is produced by severe plastic deformation (SPD) [17,18,19]. The UFG structure formation considerably increases the mechanical properties of Mg alloys. However, the effect of a UFG structure on the corrosion properties is ambiguous, which is related to the fact that SPD processing may significantly change the phase composition of alloys [20]. In its turn, the morphology and origin of secondary phases have a great effect on the corrosion behavior in Mg alloys [21,22]. Recently, this issue was carefully studied for the Mg-1Ca alloy processed by high-pressure torsion (HPT) [23].

This paper reports on the studies of the corrosion behavior of promising Mg alloys for medical applications, Mg-1%Ca and Mg-1%Zn-0.2%Ca, with a UFG structure produced by high-pressure torsion. Special attention is given to the formation of the nano-sized precipitates of second phases in these alloys and their relationship with the corrosion properties in Ringer’s solution.

## 2. Materials and Methods

The Mg-1%Ca and Mg-1%Zn-0.2%Ca alloys in the cast condition were selected for the study. The Mg-1Ca alloy billets in the cast condition with a cylindrical shape 20 mm in diameter and 100 mm in length were received from the School of Materials Science and Engineering, Peking University (China), and the Mg-1Zn-0.2Ca alloy casts with a size of 300 mm × 200 mm × 200 mm were produced at the Solikamsk Pilot Metallurgical Plant (Russia). A cylinder with a diameter of 20 mm and length of 100 mm was cut out from the casts. In order to even out the chemical composition in the sample volume and eliminate the consequences of dendritic segregation, the cylinders were heat treated in a muffle furnace at a temperature of 450 °C for 24 h with water cooling. Samples with a diameter of 20 mm and a height of 1 mm for further SPD processing were cut out from the homogenized cylinders.

In order to produce the UFG state, the homogenized samples were subjected to deformation and heat treatment (HT). At the first stage, the samples were subjected to high-pressure torsion at room temperature using an original HPT processing facility. Here, disks with a diameter of 20 mm and thickness of 1 mm were processed by high-pressure torsion under a pressure of 6 GPa with a rate of 1 rev/min. The number of revolutions during the processing was 10. At the second stage, the samples were held at temperatures of 250 °C (for the Mg-1%Ca alloy) and 300 °C (for the Mg-1%Zn-0.2%Ca alloy) for 1 h with subsequent water quenching. The fabrication and processing of the alloys are described in detail in our recent papers [24,25].

The macrostructure of the samples was studied on an Olympus GX51 (Olympus Corp., Tokyo, Japan) optical microscope. To reveal the structure, the samples were etched in a solution containing 2.5 g of picric acid, 2.5 mL of nitric acid, 5 mL of water, 50 mL of ethanol. The etching time was from 10 s to several minutes. The microstructure was analyzed using a JEM-6390 (JEOL Ltd., Tokyo, Japan) scanning electron microscope and a JEM-2100 (JEOL Ltd., Tokyo, Japan) transmission electron microscope with accelerating voltages of 10 kV and 200 kV, respectively. Thin foils were prepared by double-sided jet electropolishing on a Tenupol-5 facility using an electrolyte with the following composition: 30% nitric acid and 70% methanol. The polishing was performed at a temperature of −30 °C and a stress of 8–14 V. Due to the strain nonuniformity along the sample radius during the HPT processing, the structural studies were performed at mid-radius of the HPT-processed samples. The mean grain size obtained by optical microscopy and scanning electron microscopy (SEM) was calculated by the linear-intercept method in accordance with the standard GOST 21073.3–75, and the minimum number of grains for each state was 300. The mean grain size in the HPT-processed samples was calculated from the dark-field images obtained by transmission electron microscopy (TEM). The volume fraction of the second-phase particles (Ca_2_Mg_6_Zn_3_ and Mg_2_Ca) in the homogenized state was found in the longitudinal and cross sections using the SEM results. The volume fraction of the second-phase particles after HPT processing was estimated from the TEM images. The diffraction patterns obtained by TEM were interpreted, and the Z zone axes were calculated in accordance with the standard procedure [26,27].

X-ray diffraction analysis was performed using the diffraction patterns obtained on a Rigaku Ultima IV diffractometer in the Bragg–Brentano geometry. The diffraction patterns were taken in the continuous scanning mode with a rate of 5°/min within a range of the scattering angle 2θ from 15° to 148° using monochromatized Cu radiation generated at a voltage of 40 kV and current strength of 40 mA. The lattice parameter was calculated with the PM2K software [28].

Qualitative phase analysis was performed using the PDF-2 database of X-ray data in the PDXL software package (v. 1.8.1.0) [29].

The corrosion properties were studied by the well-known gravimetric method in accordance with ASTM G1-03-E [30]. During this procedure, the samples were fully immersed into Ringer’s solution (composition: 8.6 g/L NaCl; 0.3 g/L KCl; 0.25 g/L CaCl_2_, normal saline pH 7) and held at a temperature of 22 °C. Every 24 h, the samples were removed from the solution and weighed on an A&D GR-200 analytical balance providing measurement precision up to 0.1 mg. Prior to mass measurement, corrosion products (Mg(OH)_2_) were removed from the samples by washing in an ultrasonic bath in a washing solution with the following composition: 200 g CrO_3_, 10 g AgNO_3_, 20 g Ba(NO_3_)_2_ and 1000 mL H_2_O [30], in order to evaluate the precise weight loss. Minimum 3 samples were taken for each state.

The corrosion rate *CR* (mm/y) was calculated in accordance with ASTM G3–63592 following the formula:

(1) $$CR=\frac{87.6\left(M_{0}-M_{1}\right)}{\mathrm{St}\rho },$$

where *CR* is the corrosion rate (mm/y); S is the sample surface area, cm^2^; *M*_0_ is the initial mass (mg); *M*_1_ is the mass after immersion (mg); t is the exposure time, h; ρ is the metal density, g/cm^2^.

The mass loss in % was calculated in accordance with the formula:

(2) $$ML=\frac{\left(M_{0}-M_{1}\right)}{M_{0}}\cdot 100\%,$$

where *ML* is mass loss in %; *M*_0_ is the initial mass, mg; *M*_1_ is the mass after immersion, mg.

## 3. Results

### 3.1. Study of the Corrosion Behavior Based on Mass Loss

**Figure 1:**
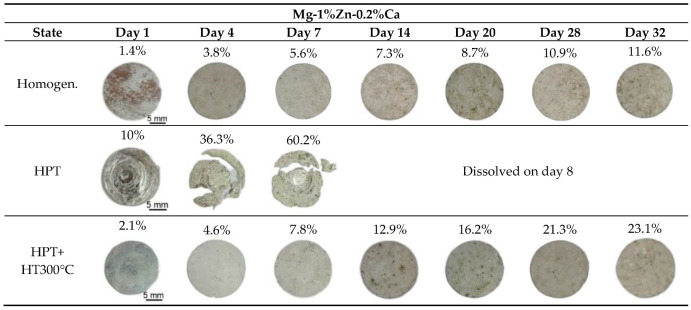
View of the Mg-1%Zn-0.2%Ca alloy samples (diameter 20 mm) in the homogenized state, after high-pressure torsion (HPT) and after HPT and heat treatment (HT) at 300 °C during corrosion tests in Ringer’s solution; the exposure time of the samples was from 1 to 32 days (optical photographs).

**Figure 2:**
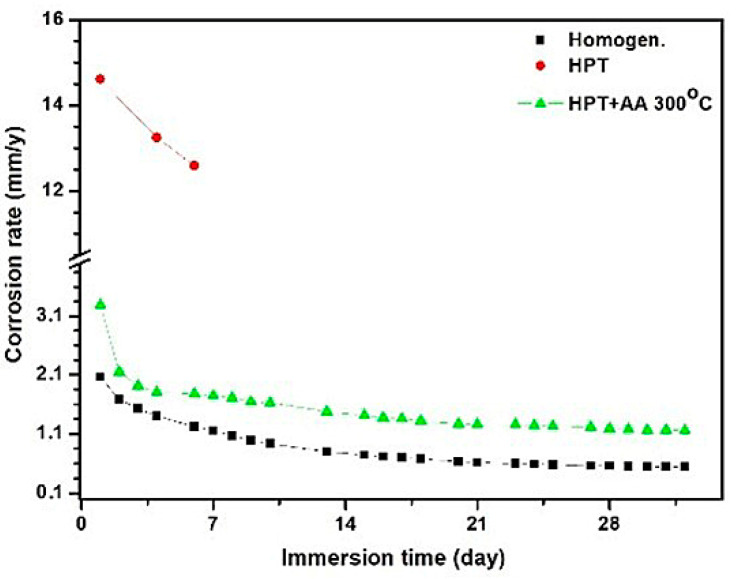
Corrosion rate of the Mg-1%Zn-0.2%Ca samples exposed in Ringer’s solution for a period of 1 to 32 days.

The results of the corrosion tests based on mass loss conducted by the gravimetric method for the Mg-1%Zn-0.2%Ca alloy are shown in Figure 1 and Figure 2 and Table 1. The appearances of the alloy samples after the first 2 days of exposure were very different. The strongest surface corrosion was observed in the HPT-processed sample: It lost 10% of its weight in the first day of the experiment (Table 1). It was found that the HPT-processed sample after 4 days of exposure started to fracture due to an intensive corrosion, and by day 7 the loss of the initial mass was 40% (Figure 1). On the surface of the samples in the homogenized state and after HPT+HT300 °C, slighter uniform corrosion was visible. The surface of the samples in the homogenized state and after HPT+HT300 °C corroded uniformly for a period of 14 days. On day 14, corrosion pits started to form on the surface of the sample after HPT+HT300 °C, i.e., pitting corrosion started to develop. The most resistant state of the Mg-1Zn-0.2Ca alloy turned out to be the homogenized state, where the corrosion rate on day 32 was 0.54 mm/y (Table 1).

Analysis of the corrosion rate of the investigated samples vividly demonstrates the intensity of the corrosion processes in the samples of the Mg-1Zn-0.2Ca alloy with different structural states (Figure 2). The HPT-processed Mg-1Zn-0.2Ca sample corroded intensively with a rate of 14.61 mm/y on day 1 of the experiment; furthermore, on day 4, the corrosion rate decreased to 13.25 mm/y, and the sample fractured. The sample after HPT+HT corroded intensively with a rate of 3.28 mm/y on day 1, but on day 4 the corrosion rate sharply decreased to 1.8 mm/y. This result indicates that in the first 4 days the force of corrosion considerably declined and continued to decline further (Figure 2). The lowest corrosion rate during the whole experiment duration was exhibited by the Mg-1Zn-0.2Ca alloy in the homogenized state.

**Figure 3:**
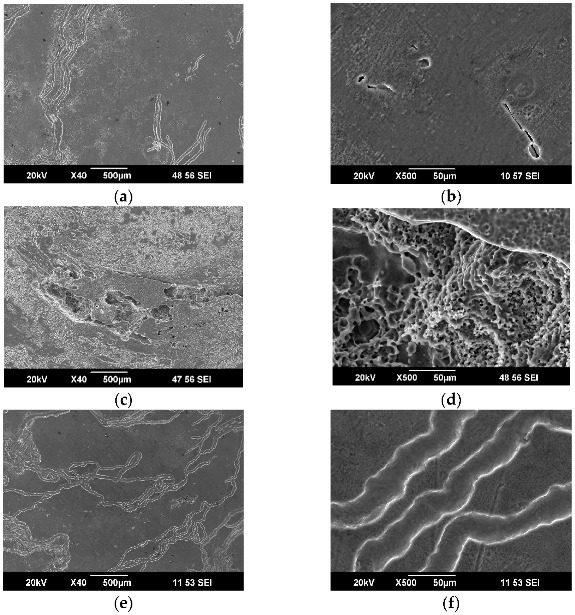
Surface structure of the Mg-1Zn-0.2Ca alloy samples: (**a**,**b**) in the homogenized state, (**c**,**d**) after HPT, (**e**,**f**) after HPT and additional HT at 300 °C, after 1 day of exposure in Ringer’s solution.

A detailed study of the sample surfaces by SEM revealed a difference in the corrosion of the investigated samples. It can well be seen that after the exposure of the HPT-processed Mg-1%Zn-0.2%Ca sample in Ringer’s solution, on day 1, deep corrosion pits formed on the surface, characterized by a high pore density (Figure 3c,d). Such corrosion pits were absent in the homogenized samples and those after HPT+HT (Figure 3a,b,e,f). Those samples were characterized by the formation of rare specific corrosion grooves.

**Figure 4:**
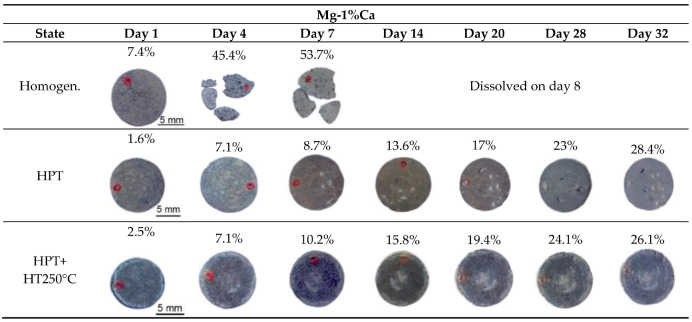
View of the Mg-1%Ca alloy samples (diameter 20 mm) in the homogenized state, after HPT and HPT+HT250 °C during corrosion tests in Ringer’s solution; the exposure time of the samples was from 1 to 32 days (optical photographs).

**Figure 5:**
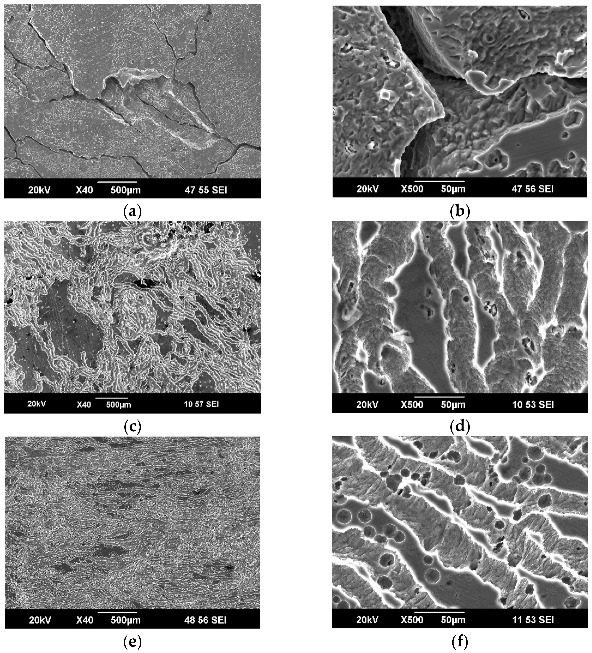
Surface structure of the Mg-1%Ca alloy samples: (**a**,**b**) in the homogenized state, (**c**,**d**) after HPT, (**e**,**f**) after HPT and additional HT at 250 °C, after 1 day of exposure in Ringer’s solution.

Furthermore, the Mg-1Ca alloy was investigated. The results of the corrosion tests based on mass loss and corrosion rate, performed by the gravimetric method, for the Mg-1Ca alloy are shown in Figure 4 and Figure 5 and in Table 2. Polished samples were immersed into Ringer’s solution, and their mass loss was investigated. In 24 h, corrosion products in the form of a white deposit formed on the surface of all the Mg-1Ca samples. It can be seen that micropits (small white dots in the photos presented in Figure 4) formed on the surface of the Mg-1Ca alloy in all the states (the homogenized state, after HPT and HPT+HT250 °C). However, an increase in the exposure time (4 and more days) of the Mg-1Ca samples in Ringer’s solution resulted in large differences in the state of the surface. The homogenized sample after 4 days of exposure in Ringer’s solution lost its integrity (Figure 4), and its weight decreased practically two-fold (Table 2). The samples processed by HPT and HPT+HT250 °C after 4 days of exposure in Ringer’s solution remained integral and had a white deposit on their surfaces.

The results demonstrate that the HPT-processed samples exhibited the best corrosion resistance. Even after 32 days of exposure in Ringer’s solution, they lost only a third of their weight (Figure 4, Table 2); the corrosion rate on day 32 was 0.54 mm/y (Table 2). The samples after HPT+HT250 °C exhibited a good corrosion resistance; the corrosion rate on day 32 of exposure was 0.67 mm/y, which is slightly worse than that of the HPT-processed samples (Figure 4).

The surface of the Mg-1Ca samples after exposure in Ringer’s solution was studied by SEM. Prior to the surface study, the samples were washed with a chemical agent to remove corrosion products.

It was found that already on day 1 of exposure in Ringer’s solution, the Mg-1Ca alloy in the homogenized state was characterized by the formation of deep pits and striations in locations where there were eutectics (Figure 5a). Eutectics in the Mg-1Ca alloy represented Mg_2_Ca particles surrounded by the Ca-depleted solid solution of Mg. According to literature data, the α-Mg–Mg_2_Ca pair has a high electrochemical activity [23]. It can be seen from the surface structure that each large corrosion pit emerges at a location of eutectics and is a result of microgalvanic corrosion caused by the α-Mg–Mg_2_Ca interaction [31]. The surface adjacent to the striations is also covered by small pits, which indicates the simultaneous intensive corrosion of both the eutectics and the matrix (Figure 5b).

Characteristic of the HPT-processed sample that exhibited the best corrosion resistance is the formation of specific grooves on the surface, as well as small pits (Figure 5c,d), at locations where presumably the Mg_2_Ca particles were present.

The surface of the samples after HPT+HT250 °C had a higher density of grooves and larger pits compared with the HPT-processed sample (Figure 5e,f).

**Figure 6:**
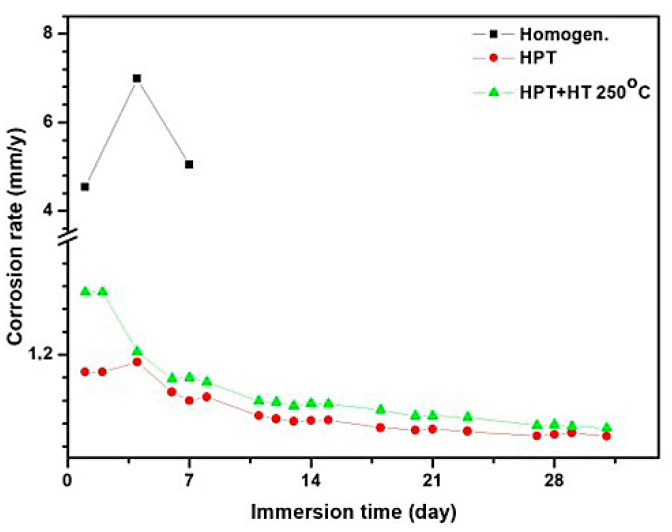
Corrosion rate of the Mg-1%Ca samples exposed in Ringer’s solution for a period of 1 to 32 days.

On the whole, the results of the study of the sample surfaces are in good agreement with the results on the corrosion rate of the samples in different states (Figure 6). It is apparent that the homogenized samples, starting from the first days of the experiment, experienced an intensive corrosion, whereas the corrosion rate in the samples after HPT and HPT+HT250 °C was several times lower. In all the cases, the corrosion rate decreased with increasing time of exposure of the samples in Ringer’s solution. Most probably, this is related to the gradual deceleration of the process due to the formation of corrosion products on the surface, as well as the attenuation of the oxidation-reduction reaction between particles and the solid solution.

### 3.2. Results of Microstructural Studies

**Figure 7:**
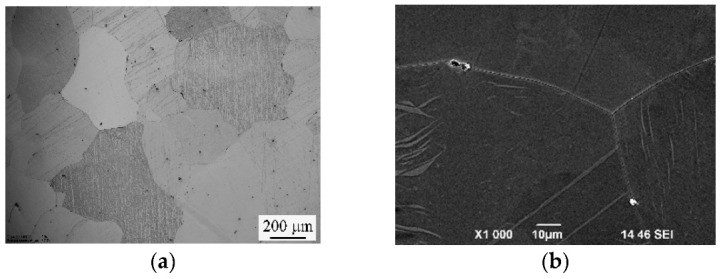

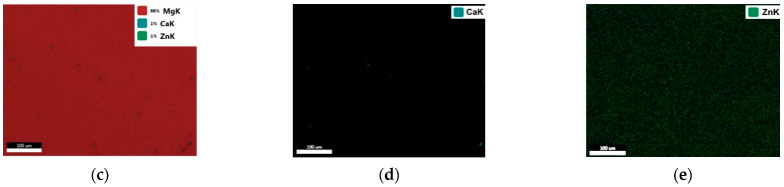
Structure of the Mg-1%Zn-0.2%Ca alloy in the homogenized state: (**a**) optical microscopy; (**b**) scanning electron microscopy (SEM); energy-dispersive spectroscopy (EDS) elemental mapping demonstrating the solute distribution: Mg, Ca, Zn (**c**), Ca (**d**), Zn (**e**).

The structure of the Mg-1Zn-0.2Ca alloy samples in the homogenized state consists of coarse solid-solution grains with a mean grain size of 270 μm (Figure 7a). Also present in grain interiors and at grain boundaries are particles of a round shape with a size of up to 4 μm with a volume fraction of below 2%. It was found in [32,33] that when the Zn/Ca ratio in atomic percent is larger than 1.2–1.4, the Ca_2_Mg_6_Zn_3_ particles are formed. In the case of our alloy Mg-1Zn-0.2Ca, the Zn/Ca ratio in atomic percent is 3.1; therefore, one may expect the formation of the Ca_2_Mg_6_Zn_3_ particles. Elemental analysis by energy-dispersive X-ray spectroscopy (EDS) of the surface of the samples in the homogenized state confirmed the presence of particles containing Ca and Zn.

**Figure 8:**
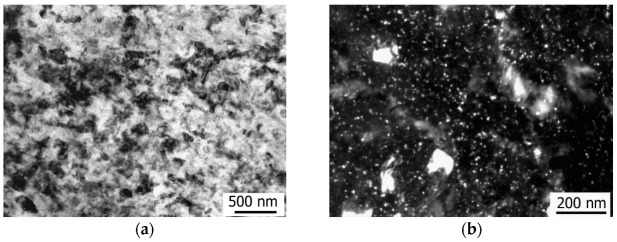
Structure of the Mg-1Zn-0.2Ca alloy after HPT processing: (**a**) bright-field image; (**b**) dark-field image showing dispersed particles.

During the HPT processing, a refined grain–subgrain structure with a mean grain size of 90 nm formed in the Mg-1Zn-0.2Ca alloy samples (Figure 8a). Most grains are characterized by a high defect density and large internal stresses. Also found in the structure of the HPT-processed samples are the fine-dispersed Ca_2_Mg_6_Zn_3_ particles with a size of 10 nm, visible as white dots in the dark-field image (Figure 8b). The volume fraction of the fine-dispersed particles is about 1%.

**Figure 9:**
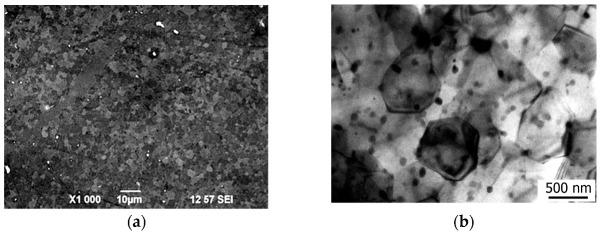

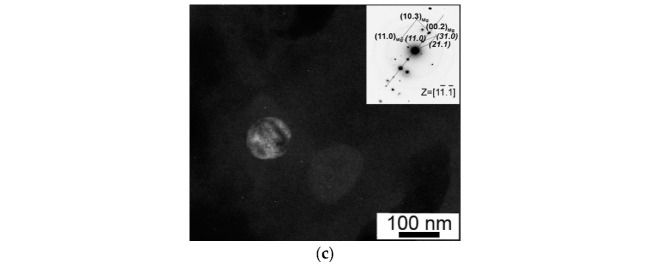
Structure of the Mg-1Zn-0.2Ca alloy after HPT+HT at 300 °C: (**a**) SEM; (**b**) transmission electron microscopy (TEM); (**c**) dark-field image of the Ca_2_Mg_6_Zn_3_ particle of a globular shape with the corresponding diffraction pattern.

The microstructure of the Mg-1Zn-0.2Ca alloy after HPT processing and additional annealing at a temperature of 300 °C is presented in Figure 9, which reveals that the average grain size increased to 4 μm, and the particle size increased to 70 nm. Analysis of the diffraction pattern obtained from a globular particle ([1$\bar{1}$.$\bar{1}$] zone axis) confirms that this particle represents the Ca_2_Mg_6_Zn_3_ phase with a hexagonal close-packed (HCP) lattice (Figure 9c). The volume fraction of the second phase Ca_2_Mg_6_Zn_3_ is about 1.5%.

**Figure 10:**
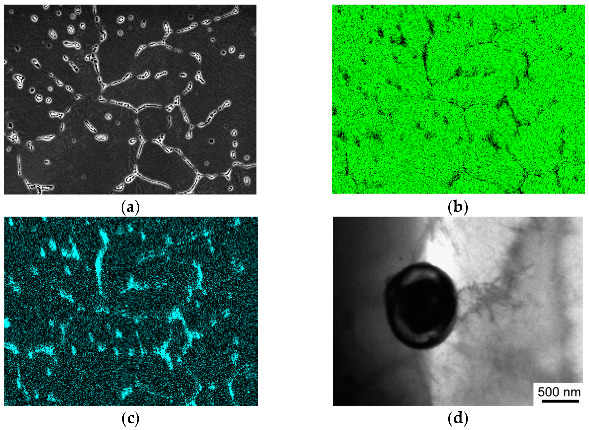
Microstructure of the Mg-1%Ca alloy in the homogenized state and elemental mapping of the alloy’s microstructure: (**a**) typical structure (SEM); (**b**,**c**) elemental mapping of the microstructure: distribution of the chemical elements Mg (**b**) and Ca (**c**); (**d**) Mg_2_Ca particle of a globular shape with the corresponding diffraction pattern in the insert (TEM).

The structure of the Mg-1%Ca alloy in the homogenized state is a solid solution (α-Mg) with the precipitation of eutectics (α-Mg+Mg_2_Ca) along the grain boundaries and in the interiors of round-shaped grains (Figure 10). EDS revealed that Ca is present in the alloy in both the eutectics and the α-matrix, and a larger content of Ca is visible in the eutectics than in the matrix (Figure 10b,c). The mean grain size is 42 µm, and coarse grains with a size of about 200 µm are observed. The volume fraction of eutectics is 5.8%. Also found in the structure are the Mg_2_Ca particles of a globular shape with a size of 600 nm (Figure 10d).

**Figure 11:**
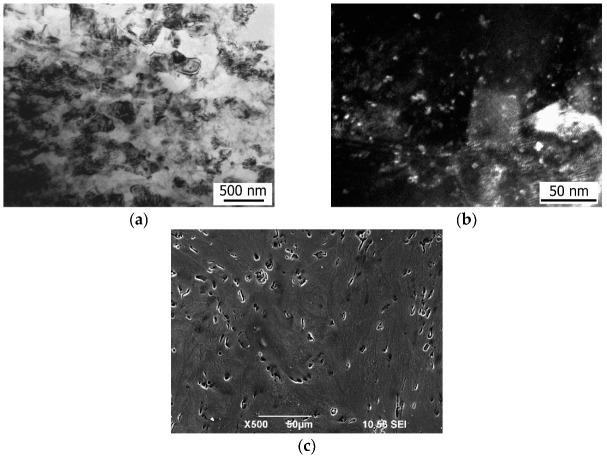
Structure of the Mg-1Ca alloy after HPT processing: (**a**) bright-field image; (**b**) view of the Mg_2_Ca particles in a dark-field image; (**c**) SEM image of the fragmented eutectics.

Study by TEM showed that HPT processing leads to strong grain refinement, and the mean grain size is 210 nm in the Mg-1Ca alloy (Figure 11a). The diffraction contrast in the bright-field and dark-field images indicates the presence of high internal stresses in the structure, induced by large shear strains under high pressures. The dispersed Mg_2_Ca particles of a round shape with a size of about 5 nm are found in the UFG structure. The Mg_2_Ca particles are visible as white dots in the dark-field image (Figure 11b). It can be concluded from the SEM images that, as a result of shear strain, during HPT processing, the eutectics became fragmented, and their volume fraction decreased to 4.1%; most probably, they partially dissolved in the solid solution under severe plastic deformation (Figure 11c).

**Figure 12:**
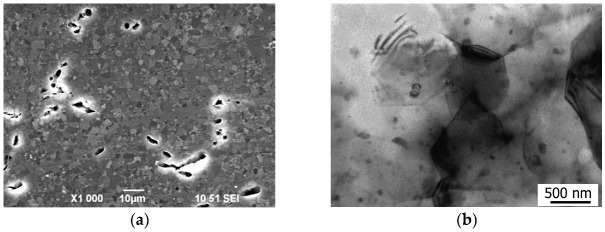
Image of the Mg-1Ca structure after HPT+HT at 250 °C: (**a**) SEM; (**b**) TEM.

The microstructural analysis of the Mg-1%Ca alloy after HPT processing and HT at 250 °C demonstrated an increase in the mean size of grains and particles (Figure 12a,b). The volume fraction of eutectics after HPT+HT at 250 °C amounts to 5.1%. According to the SEM and TEM images, the mean grain size is 1.4 µm. Study by TEM revealed a low dislocation density and the presence of thickness extinction contours. In the structure, particles with a mean size of about 70 nm, having a predominantly globular shape (Figure 12b) are found. One may assume that after the heat treatment of the HPT-processed samples, coagulation of the fine-dispersed Mg_2_Ca particles occurred as a result of the breakdown of the solid solution produced by HPT processing. X-ray analysis confirms the presence of the second phase Mg_2_Ca (Table 3). The lattice parameter of pure Mg is smaller than the lattice parameter of the Mg-1%Ca alloy samples, indicating some dissolution of Ca in the Mg lattice. After HPT processing, an even larger increase in the lattice parameters was observed, which can be explained by further dissolution of Ca in the Mg lattice during HPT processing. The volume fraction of undissolved particles in the HPT-processed samples is 4.1% (Table 3). Subsequent annealing leads to the precipitation of the Mg_2_Ca phase as a result of the breakdown of the solid solution produced by HPT processing and, correspondingly, its volume fraction increases to 5.1%.

## 4. Discussion

In this study, we have investigated the relationship between the HPT-produced structure and corrosion properties of the magnesium alloys Mg-1Zn-0.2Ca and Mg-1Ca.

It is known [32,33] that the standard electrode potential of precipitating particles follows the dependence: Ca_2_Mg_6_Zn_3_ > α-Mg > Mg_2_Ca; for example, the Mg_2_Ca particles in the Mg-1Ca alloy system have a high electrochemical activity [23]. The results of the present study demonstrate that the structural state, as well as the variation of the volume fraction and dispersion degree of particles, have a significant effect on the corrosion behavior of the magnesium alloys Mg-1Zn-0.2Ca and Mg-1Ca.

The corrosion resistance of the homogenized Mg-1Ca alloy exhibited very low values. Already on day 4, the sample fractured and lost almost a half of its mass. As it is known [32], in the Mg-1Ca alloy the Mg_2_Ca particle acts as an anode, while α-Mg acts as a cathode. In an oxidation-reduction reaction, an anode dissolves; correspondingly, in the Mg-1Ca system, the Mg_2_Ca particles will dissolve, and pitting corrosion will occur on the surface. Such large corrosion pits in the region of eutectics were observed on the surface in the homogenized state (Figure 5a,b).

Williams et al. [34] showed earlier that in Mg the points of anodic corrosion initiation propagated radially, leaving behind a corroded and blackened surface. This dark surface contained a large amounts of corrosion products. It was noted that with increasing corrosion products the corrosion rate declined considerably, and the corrosion mechanism changed from a local one to a uniform-surface one. Thus, an additional barrier in the form of corrosion products may influence the potential difference and corrosion activity in Mg alloys.

A high electrochemical activity of the α-Mg–Mg_2_Ca pair contributed to an intensive corrosion of homogenized Mg-1Ca, especially during the first 4 days when the corrosion rate increased to 7 mm/y. The rather large and extensive regions of eutectics contributed to a continuous propagation of corrosion. As a result of such active corrosion, the sample was already fractured on day 4 of the experiment.

It has been found that an efficient method for increasing the corrosion resistance of the Mg-1Ca alloy is the formation of a special structural and phase state that is produced by HPT. Such a state is characterized by a uniform distribution of refined eutectics in the volume and its partial dissolution in the solid solution. An assumption has been made that with grain refinement the width of the cathodic regions in the neighborhood of the secondary phases and at grain boundaries varies weakly, and thus, the cathode area may cover the whole grain; this contributes to a decrease in potential difference [35]. Therefore, the observed decrease in the surface potential difference ΔV between α-Mg and the Mg_2_Ca particles [23] promotes an increase in the corrosion resistance of the HPT-processed Mg-1Ca alloy. From the microstructure perspective, in the HPT-processed state, the Mg_2_Ca particles no longer form large trajectories for corrosion propagation at grain boundaries, which was characteristic of the homogenized state.

The obtained data confirm that the HPT-processed Mg-1Ca alloy has the best corrosion resistance, while an additional heat treatment somewhat reduces its corrosion resistance.

On the whole, the experiment results show convincingly that, first, large eutectic interlayers in the Mg-1Ca alloy in the homogenized state reduce its corrosion resistance and are the centers of corrosion. Second, the reason for the good corrosion resistance of the HPT-processed Mg-1Ca samples may be a change in the Ca content in the solid solution during the HPT processing. Such a nonequilibrium state may lead to a change in the potential difference of the galvanic pair Mg_2_Ca particles—solid solution. However, this hypothesis requires a detailed study of the chemical composition of the solid solution and precipitating particles at each experiment stage and finding in parallel the electrochemical potential of the particles and solid solution. These tasks will be addressed in our future works.

To support the statements made above, the data for the corrosion rate of the Mg-1Ca alloy after HPT+HT can be mentioned. It is apparent that the corrosion rate of the samples after HPT+HT considerably increased compared with the HPT-processed state, especially after 1 day of exposure in Ringer’s solution (Figure 6). At the microstructural scale, the annealing of the HPT-processed sample raised the sizes and volume fraction of the Mg_2_Ca particles from 4.1% in the HPT-processed samples to 5.1% in the samples after HPT+HT. An increase in the quantity of the Mg_2_Ca particles in the sample after HPT+HT promoted an increase in corrosion rate. In addition, the stoichiometry of the solid solution after annealing became closer to the equilibrium state, and the potentials of the galvanic pair Mg_2_Ca—solid solution should tend to the initial state. A combination of these two resulted in the development of corrosion processes in the sample after HPT+HT via the same mechanisms as in the homogenized sample of the Mg-1Ca alloy.

Comparative studies of the corrosion properties of the two alloys, Mg-1Ca and Mg-1Zn-0.2Ca, in the homogenized state, on the whole, indicate that an addition of Zn into the Mg-Ca system leads to an increase in the alloy’s corrosion stability.

In the ternary Mg-Zn-Ca alloys, a relatively low corrosion rate is explained by the enrichment in zinc of the matrix phase reducing the potential difference between the matrix and the Ca_2_Mg_6_Zn_3_ ternary particle [36]. The predominant corrosion mechanism in the Mg-Zn-Ca ternary alloys is controlled by microgalvanic coupling between the ternary phase and matrix. Thus, the Mg-Ca-Zn alloys with a small fraction of ternary particles have few cathodic sites and a relatively low corrosion rate [36]. Therefore, due to a relatively small quantity of the Ca_2_Mg_6_Zn_3_ particles in the homogenized Mg-1Zn-0.2Ca alloy (Figure 2), the galvanic reaction between a particle and the matrix cannot be as strong as in the homogenized Mg-1Ca alloy with many large corrosion sites in the form of the Mg_2_Ca particles.

As expected, the HPT processing of the homogenized Mg-1Zn-0.2Ca alloy resulted in the alloy’s microstructure refinement and the formation of very fine Ca_2_Mg_6_Zn_3_ particles with a size of about 10 nm. However, unlike in the Mg-1Ca alloy, this sharply decreased the corrosion resistance of the Mg-1Zn-0.2Ca alloy. As it is known [32], in the Mg-1Zn-0.2Ca alloy the Ca_2_Mg_6_Zn particles act as a cathode, while α-Mg is an anode. Correspondingly, in the Mg-1Zn-0.2Ca sample, a continuous corrosion at grain boundaries and crystalline structure defects should prevail. The HPT processing of the Mg-1Zn-0.2Ca alloy resulted in a considerable increase in the fraction of nonequilibrium boundaries and dislocation density, which, respectively, increased the number of corrosion centers and, on the whole, catalyzed corrosion processes. This assumption is well confirmed by the very high corrosion rate of the HPT-processed samples (Figure 2) and the high quantity of corrosion sites on the surface of the UFG Mg-1Zn-0.2Ca alloy already after a one-day exposure in Ringer’s solution (Figure 3c,d). In addition, one may assume that during the HPT processing of the Mg-1Zn-0.2Ca alloy, the stoichiometry of the solid solution could also deviate from the equilibrium value, and, correspondingly, the potential difference of the galvanic pair Ca_2_Mg_6_Zn—solid solution could change for the worse.

A considerable improvement of the corrosion properties of the UFG Mg-1Zn-0.2Ca alloy was achieved by influencing grain boundaries and the intragranular volume by means of recrystallization annealing. As a result of the annealing, grain size increased from 90 nm to 4 μm (which means that the density of boundaries sharply decreased); dislocation density decreased; and the sizes of the Ca_2_Mg_6_Zn_3_ particles increased by a factor of 7, from 10 nm to 70 nm. As a consequence, the corrosion rate of the samples after HPT+HT significantly declined and was comparable to that of the homogenized samples. This excellent result vividly demonstrates the possibility of controlling efficiently the corrosion mechanisms and rate in Mg-Zn-Ca alloys by means of producing specific structures due to a combination of heat treatment and HPT processing.

Studies of the surface of the Mg-1Zn-0.2Ca and Mg-1Ca alloy samples after corrosion tests in Ringer’s solution reveal a common regularity. In all the cases, a good corrosion resistance was exhibited by the samples that had specific corrosion grooves on the surface and did not have deep corrosion pits. A low corrosion resistance was exhibited by the samples that had large corrosion sites (homogenized Mg-1Ca, HPT-processed Mg-Zn-Ca), which stimulated the propagation of corrosion into the sample’s depth and sample fracture.

The magnesium alloys Mg-1Zn-0.2Ca and Mg-1Ca exhibited totally different corrosion properties after HPT processing. The HPT-processed Mg-1Ca alloy exhibited the best corrosion resistance, sufficient for the use as an implant material (the required corrosion rate is not more than 1 mm/y). The HPT-processed Mg-1Zn-0.2Ca alloy exhibited the worst corrosion resistance and fractured on day 4 of the experiment. The revealed paradox of the corrosion properties of these alloys cannot be accounted for solely by grain refinement and nonequilibrium boundaries. It is assumed that the morphological parameters of the particles, as well as a change in the potential difference of the galvanic pairs “particle–matrix” during the alloy’s transition to the nonequilibrium state, may influence the activity of corrosion mechanisms. This hypothesis will be examined in more detail in future research, including the VPD (Volta potential difference) between Mg an Mg_2_Ca/Ca_2_Mg_6_Zn_3_ particles.

Rather strict requirements are imposed on bioresorbable Mg alloys for producing medical implants in terms of their strength properties (ultimate tensile strength at least 300 MPa) and corrosion resistance (typically not more than 1 mm/y) [2,5]. Enhancement of the strength properties through grain refinement appears to be the most relevant approach for Mg alloys. For strengthened UFG Mg alloys, the preservation and enhancement of their corrosion resistance remains an important task. The present work has shown that, due to the formation of a special structural and phase state, the produced samples exhibited a good corrosion resistance: for Mg-1%Ca after HPT+HT250 °C, the corrosion rate is ~0.67 mm/y, and for Mg-1%Zn-0.2%Ca after HPT+HT300 °C, the corrosion rate is ~1.16 (day 32 of the experiment). These values are close to the required values of corrosion resistance. The presented results demonstrate possible paths for improving the corrosion properties of Mg alloys and achieving a set of properties that meet present-day requirements to bioresorbable materials.

Based on literature data and the results obtained in the present study, it can be stated that the principles of the controlled biodegradation of Mg alloys should take into account both changes in structural elements, including the stoichiometry of the solid solution and particles, and changes in their potential difference. Such a multilevel dependence observed after HPT and HT treatments opens up a great variability of the “structure–properties” states.

## 5. Conclusions

The results of this research show that HPT processing has a great effect on the corrosion behavior of these alloys. It has been found that in the homogenized Mg-1Ca alloy, corrosion fracture occurs via the pitting corrosion mechanism with a rate of 7 mm/y (day 4 of the experiment) with the formation of large pits in the areas where eutectic interlayers are located. The formation, during the HPT of the Mg-1Ca alloy, of a special structural and phase state characterized by a uniform distribution of refined eutectics in the volume and its partial dissolution in the solid solution provides an almost six-fold decrease in corrosion rate, to 1.13 mm/y on day 4 of the experiment.

It has been experimentally proven that the corrosion fracture of the Mg-1Zn-0.2Ca alloy in the homogenized state is controlled by the continuous mechanism of corrosion with a rate of 1.39 mm/y (day 4 of the experiment). The HPT processing of the Mg-1Zn-0.2Ca alloy produced a nonequilibrium structure having a high density of deformation-induced grain boundaries with a mean grain/subgrain size of 90 nm and Ca_2_Mg_6_Zn_3_ particles 10 nm in size. The Mg-1Zn-0.2Ca alloy with such a nonequilibrium fine-grained structure exhibited a very high corrosion rate of 13.25 mm/y (day 4 of the experiment). The annealing of the UFG Mg-1Zn-0.2Ca alloy at 300 °C evened out the chemical composition of the solid solution and led to the recrystallization of grains and an increase in grain size to 4 μm and the Ca_2_Mg_6_Zn_3_ particle size to 70 μm. This provided a considerable improvement in the corrosion resistance of the Mg-1Zn-0.2Ca alloy.

On the whole, although SPD processing significantly increases mechanical properties, as the data of the present study show, its effect on the corrosion behavior may vary. This is related to the fact that SPD processing not only refines the grain structure but also influences the morphology of second-phase precipitates, which, in turn, have a significant effect on the corrosion behavior. Therefore, not only the choice of an alloy composition is required for medical use but also the choice of processing technique and regimes, which should be a special task for the practical applications of Mg alloys.

## Figures and Tables

**Table 1:** Mass loss and corrosion rate of the Mg-1Zn-0.2Ca alloy during corrosion tests in Ringer’s solution.

Immersion Time (day)	Mass Loss (%)			Corrosion Rate (mm/y)		
	Homogen.	HPT	HPT+HT300 °C	Homogen.	HPT	HPT+HT300 °C
1	1.4	10	2.1	2.06	14.61	3.28
4	3.8	36.3	4.6	1.39	13.25	1.800
7	5.6	60.2	7.8	1.15	12.57	1.74
14	7.7		13.5	0.74		1.41
20	8.7		16.2	0.63		1.26
28	10.9		21.3	0.56		1.18
32	11.6		23.1	0.54		1.16

**Table 2:** Mass loss and corrosion rate of the Mg-1%Ca alloy during corrosion tests in Ringer’s solution.

Immersion Time (day)	Mass Loss (%)			Corrosion Rate (mm/y)		
	Homogen.	HPT	HPT+HT250 °C	Homogen.	HPT	HPT+HT250 °C
1	7.4	1.6	2.5	4.54	1.04	1.74
4	45.4	7.1	7.1	7	1.13	1,22
7	57.3	8.7	10.2	5	0.8	0.98
14		13.6	15.8		0.62	0.77
20		17	19.4		0.54	0.66
28		23	24.1		0.69	0.58
32		26.4	28.1		0.57	0.67

**Table 3:** Values of the lattice parameters *a* and *c* and the volume fraction of Mg_2_Ca in the Mg-1%Ca alloy in various states.

State of the Mg-1%Ca Alloy	Lattice Parameter, A	Volume Fraction of Mg_2_Ca, %
Homogenized	*a* = 3.2116 ± 0.00004 *c* = 5.2115 ± 0.00007	5.8 ± 0.3
Homogenized + HPT	*a* = 3.21183 ± 0.00004 *c* = 5.21197 ± 0.00006	4.1 ± 0.2
Homogenized + HPT + HT at 250 °C	*a* = 3.21039 ± 0.00006 *c* = 5.21148 ± 0.00009	5.1 ± 0.3

## Data Availability

Not applicable.

## References

[1] Zhao D., Witte F., Lu F., Wang J., Li J., Qin L. (2017). Current status on clinical applications of magnesium-based orthopaedic implants: A review from clinical translational perspective. Biomaterials.

[2] Yang Y., Zhou J., Chen Q., Detsch R., Cui X., Jin G., Virtanen S., Boccaccini A.R. (2019). In vitro osteocompatibility and enhanced biocorrosion resistance of diammonium hydrogen phosphate-pretreated/poly (ether imide) coatings on magnesium for orthopedic application. ACS Appl. Mater. Interfaces.

[3] Zheng Y. (2015). Magnesium Alloys as Degradable Biomaterials.

[4] Yin D.-S., Zhang E.-L., Zeng S.-Y. (2008). Effect of Zn on mechanical property and corrosion property of extruded Mg-Zn-Mn alloy. Trans. Nonferrous Met. Soc. China.

[5] Ding W. (2016). Opportunities and challenges for the biodegradable magnesium alloys as next-generation biomaterials. Regen. Biomater..

[6] Song G., Atrens A. (2003). Understanding magnesium corrosion: A framework for improved alloy performance. Adv. Eng. Mater..

[7] Raman R.S.K., Choudhary L. (2013). Cracking of magnesium-based biodegradable implant alloys under the combined action of stress and corrosive body fluid: A review. Emerg. Mater. Res..

[8] Choudhary L., Raman R.S.K. (2012). Magnesium alloys as body implants: Fracture mechanism under dynamic and static loadings in a physiological environment. Acta Biomater..

[9] Song G., Song S. (2007). A possible biodegradable magnesium implant material. Adv. Eng. Mater..

[10] Gu X., Zheng Y., Cheng Y., Zhong S., Xi T. (2009). In vitro corrosion and biocompatibility of binary magnesium alloys. Biomaterials.

[11] Hofstetter J., Rüedi S., Baumgartner I., Kilian H., Mingler B., Povoden-Karadeniz E., Pogatscher S., Uggowitzer P.J., Löffler J.F. (2015). Processing and microstructure–property relations of high-strength low-alloy (HSLA) Mg-Zn-Ca alloys. Acta Mater..

[12] Seong J.W., Kim W.J. (2015). Mg-Ca binary alloy sheets with Ca contents of ≤1 wt.% with high corrosion resistance and high toughness. Corros. Sci..

[13] Jin Y., Blawert C., Feyerabend F., Bohlen J., Silva C.M., Gavras S., Willumeit R.S.R. (2019). Time-sequential corrosion behaviour observation of micro-alloyed Mg0.5Zn-0.2Ca alloy via a quasi-in situ approach. Corros. Sci..

[14] Li Z., Gu X., Lou S., Zheng Y. (2008). The development of binary Mg–Ca alloys for use as biodegradable materials within bone. Biomaterials.

[15] Merson D., Brilevski A., Myagkikh P., Tarkova A., Prokhorikhin A., Kretov E., Frolova T., Vinogradov A. (2020). The functional properties of Mg–Zn–X biodegradable magnesium alloys. Materials.

[16] Bakhsheshi R.H.R., Abdul K.M.R., Idris M.H., Farahany S. (2012). Relationship between the corrosion behavior and the thermal characteristics and microstructure of Mg–0.5Ca–xZn alloys. Corros. Sci..

[17] Valiev R.Z., Islamgaliev R.K., Alexandrov I.V. (2000). Bulk nanostructured materials from severe plastic deformation. Prog. Mater. Sci..

[18] Lowe T.C., Valiev R.Z., Tiwari A., Nordin A.N. (2014). Frontiers of bulk nanostructured metals in biomedical applications. Advanced Biomaterials and Biodevices.

[19] Kulyasova O.B., Islamgaliev R.K., Zhao Y., Valiev R.Z. (2015). Enhancement of the mechanical properties of an Mg–Zn–Ca alloy using high-pressure torsion. Adv. Eng. Mater..

[20] Valiev R.Z., Straumal B., Langdon T.G. (2022). Using severe plastic deformation to produce nanostructured materials with superior properties. Annu. Rev. Mater. Res..

[21] Zhang C., Guan S., Wang L., Zhu S., Chang L. (2017). The microstructure and corrosion resistance of biological Mg–Zn–Ca alloy processed by high-pressure torsion and subsequently annealing. J. Mater. Res..

[22] Zhang C.Z., Zhu S.J., Wang L.G., Guo R.M., Yue G.C., Guan S.K. (2016). Microstructures and degradation mechanism in simulated body fluid of biomedical Mg–Zn–Ca alloy processed by high pressure torsion. Mater. Des..

[23] Parfenov E.V., Kulyasova O.B., Mukaeva V.R., Mingo B., Farrakhov R.G., Cherneikina Y.V., Yerokhin A., Zheng Y.F., Valiev R.Z. (2020). Influence of ultra-fine grain structure on corrosion behaviour of biodegradable Mg-1Ca alloy. Corros. Sci..

[24] Kulyasova O.B., Islamgaliev R.K., Lin H.C., Yilmazer H. (2021). Microstructure and Mechanical Properties of the UFG Magnesium Alloy Mg-1%Ca. Mater. Sci. Forum.

[25] Mukaeva V.R., Kulyasova O.B., Farrakhov R.G., Parfenov E.V. (2019). Mechanical properties and corrosion behavior of Mg-1Zn-0.2Ca alloy with various grain size. IOP Conf. Ser.: Mater. Sci. Eng..

[26] Hirsch P., Howie A., Nicholson R.B., Pashley D.W., Whelan M.J. (1977). Electron Microscopy of Thin Crystals.

[27] Fultz B., Howe J.M. (2013). Transmission Electron Microscopy and Diffractometry of Materials.

[28] Leoni M., Confente T., Scardi P. (2006). PM2K: A flexible program implementing Whole Powder Pattern Modelling. Z. Krist. Suppl..

[29] Rigaku C. (2010). Integrated X-Ray Powder Diffraction Software PDXL. Rigaku J..

[30] (2003). Standard Practice for Preparing, Cleaning, and Evaluating Corrosion Test Specimens.

[31] Li H.X., Qin S.K., Yang C.L., Ying Z.M., Wang J., Yun J.L., Zhang J.S. (2018). Influence of Ca addition on microstructure, mechanical properties and corrosion behavior of Mg-2Zn alloy. China Foundry.

[32] Zhang E., Yang L. (2008). Microstructure, mechanical properties and bio-corrosion properties of Mg–Zn–Mn–Ca alloy for biomedical application. Mater. Sci. Eng. A.

[33] Tong L.B., Zheng M.Y., Cheng L.R., Zhang D.P., Kamado S., Meng J., Zhang H.J. (2015). Influence of deformation rate on microstructure, texture and mechanical properties of indirect extruded Mg–Zn–Ca alloy. Mater. Charact..

[34] Williams G., McMurray H.N., Grace R. (2010). Inhibition of magnesium localised corrosion in chloride containing electrolyte. Electrochim. Acta.

[35] Miyamoto H. (2016). Corrosion of ultrafine-grained materials by severe plastic deformation, an overview. Mater. Trans..

[36] Zander D., Zumdick N.A. (2015). Influence of Ca and Zn on the microstructure and corrosion of biodegradable Mg–Ca–Zn alloys. Corros. Sci..

